# Synthesis and characterization of silver substituted strontium phosphate silicate apatite using solid-state reaction for osteoregenerative applications

**DOI:** 10.1080/21655979.2021.1899670

**Published:** 2021-04-05

**Authors:** Dong Chen, Jingxin Zhao, Xin Jiang

**Affiliations:** Department of Orthopaedics, China-Japan Friendship Hospital, Beijing, China

**Keywords:** Strontium phosphosilicate, silver, apatites, silica, osseointegration

## Abstract

Strontium phosphosilicate is one of the fastest-growing apatite in bone regeneration application due to the presence of strontium and silica components in the parent materials. However, bacterial infections cause setbacks to the bone regeneration process often leading to surgical revisions, and is a big issue that needs to be addressed. Silver on this front has proven to be a great substituent as seen in the case of calcium phosphate–based ceramics that addresses the bactericidal properties of a biomaterial. Apatite strontium phosphosilicate substituted with a stoichiometric amount of silver as a dopant was synthesized using a high-temperature solid-state reaction. The phase formation was characterized by XRD and FT-IR coupled with morphological features visualized using Electron Microscopy. Antibacterial properties were investigated quantitatively using Colony-forming unit method against both Gram-positive as well as Gram-negative bacteria. Cytotoxicity assay was performed against MG-63 Cell lines and it showed excellent biocompatibility at 25ug/ml with optimal doping of 2% silver. Further, apatite seeding and formation were characterized after immersion in simulated body fluid solution which showed apatite phase formation initiated after 4 days of treatment characterized by XRD and FT-IR studies. This apatite formation was also visualized and confirmed using SEM.

## 1. Introduction

The apatite superfamily has a wide variety of compounds that serve a vibrant range of applications. Hydroxyapatite, one such member has been long used to attend bone tissue regeneration studies. Despite superior biocompatibility, these calcium phosphate ceramics fail to address some key issues that have been speculated and explicitly reported to cause complications in the bone regeneration process [1]. One such major concern arises out of bacterial colonization post-implant surgery that is bound to bring complications that might lead to surgical revisions despite the administration of antibiotic drugs. To address this issue, researchers tried and succeeded in substituting metals to the crystal structure of Ca–P ceramics that eventually lead to the generation of the targeted antibacterial environment leading to the prohibition of any colonization and inhibition of the same if any [2,3]. These metals such as zinc and silver have been reported by biologists to have a specific pathway that attacks the bacterial cells and hence rules out any harm to normal human cells and tissues. However, it must be noted, that an uncontrolled release of these metal ions or nanoparticles tends to interfere with normal metabolic pathways of healthy cells and as a result may lead to toxic effects over healthy cells when administered in higher concentrations [4,5].

The structural features of P6_3_/M space group apatites are what makes them ideal in the bone regeneration process. With a general formula **M_10_(XO_4_)_6_Z_2_** and hexagonal bipyramidal structure, these apatites feature a tunnel-shaped form that facilitates the interaction of M as well as the X component with the body fluid that dramatically enhances the bone regeneration capabilities with the right composition. Mostly, the M component is a divalent metal, XO_4_ a trivalent or tetravalent anion, such as phosphate or silicate and Z a functional anion, such as F^−^ in fluorapatite, Cl^–^ in chlorapatite [6–8]. The parent material under study here has strontium at the M site and phosphate and silicate at the X site. Studies by Anjaneyulu et al. have shown the bone regenerative capabilities of the material in vitro as well as states the charge compensation as the mechanism of coexistence of phosphate as well as silicate components at X site maintaining the structural integrity contrary to the speculation of silicate being the Z component [9].

Strontium has a long history of being used as a dietary supplement targeted at bone health [10]. It constitutes an important component of bone composition and is involved in most of the native bone metabolism activities [11]. Several medicines such as Strontium Renalate have been well established as a curative option for maintaining skeletal integrity and regenerative processes [12,13]. There is biochemical evidence, that strontium mostly works by using and stimulating the receptors of calcium in the metabolic process that leads to favorable osteogenic activity thereby leading to an accelerated rate of bone tissue regeneration. The most important feature of this process is an elevated rate of bone cells depositions by osteoblast which increases the bone mass at the damaged tissue site. [14,15]

Silica, on the other hand, has a multifaceted effect on the bone regeneration process and has even been reported to exhibit a mild inhibitory effect on bacterial colonization. Silica-based materials have been found to bear bonding sites over the surface that readily interact with proteins as well as cells responsible for the osteogenic activity and hence stimulate bone formation better and faster [16,17]. The most prominent cells that are affected by silica are osteoblasts and fibroblasts. These cells have been established as the key components that are required for bone formation, mineralization and manifestation of Collagen I which is one of the key components of the bone [18,19]. It was already known about the bone mass on account of osteoblast cells that was confirmed by several researchers over ALP expression, but the role of silica in bone mineralization was confirmed with the identification of stimulatory effect over NF-kB pathways [20].

The native tunnel-shaped hexagonal rod-like structure of the parent material features mobile X ions and hence provides a dual surface for the interaction of M as well as X components. This cumulative interaction of strontium and silica for obvious reasons be suitable for stimulation in the bone regenerative application. However, it must be noted that silica-based materials have been reported to mildly inhibit bacterial infections. Hence, the authors have proposed to dope the M component of parent material with silver as an antibacterial agent in the parent material.

Silver is a well-established and aggressive antibacterial meta that turns toxic at high concentrations [21,22]. However, researchers have found that silver even at low concentrations can bring about good antibacterial effects on account of the oligodynamic effect [23]. The bacterial apoptosis brought about by silver is two-phased where the first phase is caused by cellular degeneration and loss in outer membrane integrity while the second phase of inhibition is brought about by the effects generated by preliminary apoptosis and enzymatic imbalance in the extracellular matrix. Most of the cellular degeneration is caused by the direct attack of the silver ions on the bacterial membrane [24]. This leads to a loss in the structural integrity of the same and often loss of cytoplasmic components. In some cases, bacterial metabolism is blocked on account of changes brought about at the cellular membranes. The loss of protein motive force (PMF) or change in overall morphology leads to the arrest of ATP channels that are crucial for bacterial survival thereby causing inhibition in bacterial growth [25]. The PMF acts as a major gradient in flow of energy that governs the functioning of ATP channel. Disruption in the PMF leads to alterations in gradient or flow of energy that inhibits the ATP channels and hence the production of ATP thereby inhibiting the cell division process in bacteria. Moreover, it has also been established that silver acts in a similar fashion in both ionic as well as nanoparticle form [26].

The role of silver in osteogenic activities is not well established but there is very scarce biochemical evidence that point possibilities of the same. Mesenchymal stem cells, one of the most prominent cells that have been linked to bone regeneration show some promising activity that points toward silver as a probable agent that helps in bone regeneration. MSCs were found to be proliferated at an elevated rate in presence of silver ions on account of the upregulation of IL-8 genes [27]. Moreover, marker genes such as CBFA-1 were also identified in presence of silver ions otherwise absent by Zhang et al. which are associated with osteoblastic differentiation. [28] Not only this, TGF-b1 and phosphorylated SMAD-5 markers, associated with the formation of callus and granulation tissue out of fibroblasts [29]. The identification of these makers points toward respective signaling induced within cells that leads to osteogenic and chondrogenic differentiation of MSCs.

The authors for the first time have successfully substituted silver in the strontium phosphate silicate crystals using a solid-state reaction. Several researchers have successfully used the same pathway and reported stable pure as well as substituted strontium phosphate silicate and studied accordingly. Four hierarchically increasing substitution was made to the molar percentage to identify the optimized level of substitution possible in the structure of apatite over all the parameters. A pure phase strontium phosphate silicate was also synthesized to compare the doping and effects brought about by the same. The authors aim to establish strontium phosphosilicate as a substitute for HAP which itself is an apatite. The availability of calcium at the damaged site and the presence of bone regeneration stimulating components such as strontium and silica opens several doors to counter inhibition of bone proliferation with excess calcium to count a few. The addition of silver and its biocompatibility evaluation further adds antibacterial functions to the parent material that is aimed at reduction of antibiotic administration as well as rule out surgical revisions due to bacterial colonization.

## 2. Materials and methods

### 2.1 Synthesis of strontium phosphate silicate powders

The powder samples of the phosphate silicate apatites Sr5−_*x*_Ag_*x*_(PO_4_)_2_(SiO_4_) (*x* = 0, 0.1, 0.2, 0.3, 0.4) were prepared by a conventional solid-state synthesis. Analytical grade strontium carbonate, silicon dioxide, ammonium dihydrogen phosphate and silver nitrate were weighed and taken accordingly as precursors. Sr/P+ Si ratio of the apatite was fixed at 1.67 with Sr source constituting 0.5 M, ammonium dihydrogen phosphate as a source of (PO)4 fixed at 0.2 M and silicon dioxide acting as Si source at 0.1 M. The reaction mixtures were separately preheated in hot air oven before the reaction process to remove any moisture followed by homogenization. This was followed by heating the reaction mixtures at 350°C for 2 hours to remove any surplus moisture followed by intimate fine grinding of the powders. These powder reaction mixtures were sintered at 800°C for a dwelling period of 5 hours.

### 2.2 Powder characterization

The X-ray diffraction phase analysis of the sintered powders was performed using a diffractometer (Panalytical X’pert^3^ MRD) with Cu Kα radiation (*λ* = 1.54056A°) produced at 40 kV and 30 mA to scan the diffraction angles (2*θ*) between 10° and 80° with a step size of 0.04° 2*θ* per second. Indexing of the phases was done using ICDD Card ‘21–1187ʹ for strontium phosphate silicate. The FT-IR analysis of powders was performed in transmission mode using an FT-IR spectrophotometer (Thermo Nicolet, Avatar 330, USA) in the spectral range from 4000 to 400 cm^−1^ by the KBr method (0.01%). The structural features of the powders were characterized and visualized using a high-resolution FE-SEM (Zeiss GeminiSEM 300) via sputtering of powders over the conductive surface.

### 2.3 Hemolytic assay

Hemolytic assay of the powders was carried out in accordance with the ASTM F-756-00 guidelines [30]. Fresh human blood was collected from volunteers and stored with anticoagulant and stored at 35°C. The blood was further diluted with sterilized physiological saline and stored in the incubator at 37°C for half an hour. Test extract from the samples was prepared by mixing 1 mg/ml sample in saline and incubated in a shaking incubator for 15 minutes. After the incubation, 9 ml of blood and 1 ml of test extract were mixed together in respective tubes and incubated for 60 minutes. The control tubes of Distilled water and Saline were incubated for 30 minutes at body temperature. The absorbance was measured using UV–Vis scanning spectrophotometer (Shimadzu UV-1800) at 545 nm and hemolysis ratio (*Z*) was calculated using the following formula:

$$Z=\frac{Dt-Dnc}{Dpc-Dnc}\times 100\%$$

Where D_t_ is the intensity of the sample, D_nc_ of Negative control and D_pc_ of positive control.

### 2.4 Antibacterial assay of Sr_5_(PO_4_)_2_SiO_4_

Quantitative antibacterial assay of the samples was performed using the colony-forming unit (CFU) counter method over both Gram Positive as well as Gram-negative bacteria. For the assay, bacterial cell cultures were obtained, inoculated in distilled water and mixed well. A series of serial dilution was carried out and the obtained bacterial culture was stored in a CO_2_ incubator. The pre-prepared test extracts of the sample stored at 37°C were taken and added to the respective tubes. The test samples were then sealed and incubated at 40°C in CO_2_ incubator for 4 hours. Following incubation, the test samples were again serial diluted till 10^−5^ and inoculated using the standard spread plate method, and incubated in a CO_2_ incubator for 24 hours. The thus-obtained colonies were counted using a digital colony counter and the CFU/ml was calculated using the following formula.

$$\begin{array}{rl} & CFU/mL\\ & \frac{Number\;of\;Colonies\;obtained\;x\;Dilution\;Factor}{Volume\;of\;Sample\;plated} \end{array}$$

### 2.5 Cytotoxicity assay

Cytotoxicity assay of the powdered samples was carried out in accordance with the method described earlier from the literature. The cultured MG 63 cell lines were used for the following procedure with the pre-prepared test extracts of the samples. The test extracts were seeded with cell lines at a concentration of 1 × 10^4^ cells in the well plate for 72 hours. The cells were washed with serum-free media and further incubated for 24 hours following which the culture media was removed from the cells. 0.5 mg/ml MTT prepared in 1x PBS was added to cells and incubated at 37°C for 4 hours which was followed by removal of MTT and washing with PBS. The as-formed crystals were dissolved in DMSO and the developed color intensity was evaluated at 570 nm. The percentage of cell viability was elucidated with respect to OD values of control using the following calculation:

$$Cell\;Viability\;\left(\%\right)=\frac{Intensity\;of\;Sample}{Intensity\;of\;Control}\times 100$$

### 2.6 Simulated body fluid assay

Reagents from Sigma Aldrich were used to formulate the simulated body fluid as described by Kokubo et al. [31] The sintered powder samples were immersed in SBF solution at a composition of 10 ml/10 mg for 4 and 7 days. The SBF solution was changed every 24 hours without losing any powder samples. After completion of the treatment period, samples were gently rinsed with deionized water, followed by drying at room temperature according to the literature. Diffraction and IR data of the treated samples were recorded and plot along with the untreated sample. Further morphological changes were visualized using SEM [32].

## 3. Results

Strontium phosphosilicate was characterized structurally to detect phase formation, and its hypothesized morphology was found to be needle-shaped unlike Hexagonal bipyramidal P63/m group. Biocompatibility studies covering RBCs, MG-63 cell lines and bacteria cumulatively show that 2X is optimum concentration of silver substitution. Structural features were examined by the diffraction data with respect to the ICDD database and FTIR. The morphology of optimized sample was examined by FE-SEM. In vitro bioactivity and biocompatibility studies were carried out using Hemolytic assay, CFU-Colony-formingbased antibacterial studies and MTT based cytotoxicity assay. The bone regenerative effects were characterized by immersing the samples in simulated body fluid (SBF) solution and the changes were investigated after characterizing using XRD and FTIR. Morphological changes were visualized using SEM images and elaborated.

### 3.1. Structural

The diffraction peaks depicted in Figure 1 show sharp diffraction peaks confirming the crystal structure. The peaks when compared with the ICDD data cards show additional minor secondary peaks of strontium phosphate and strontium silicate. Apart from this, no visible changes are observed throughout. Zooming in to the plot, it was observed that the major peaks tend to shift toward greater 2*θ* values as the dopant concentration is increased and the shift is almost in line with each other (Figure 1a). The average crystallite size was deduced to be around 40 nm of the pure phase along with the substituted samples (Table 1).

**Table 1.** Grain size of samples calculated via the Scherrer equation from diffraction data

Sample	FWHM	FWHM (Radians)	Size (Angstrom)	Size (nm)
Pure	0.18856	0.0032	436.74	43.67
1x	0.18976	0.0033	434.07	43.41
2x	0.20127	0.0035	409.27	40.93
3x	0.20189	0.0035	408.1	40.81
4x	0.23332	0.004	353.18	35.32

**Figure 1.**
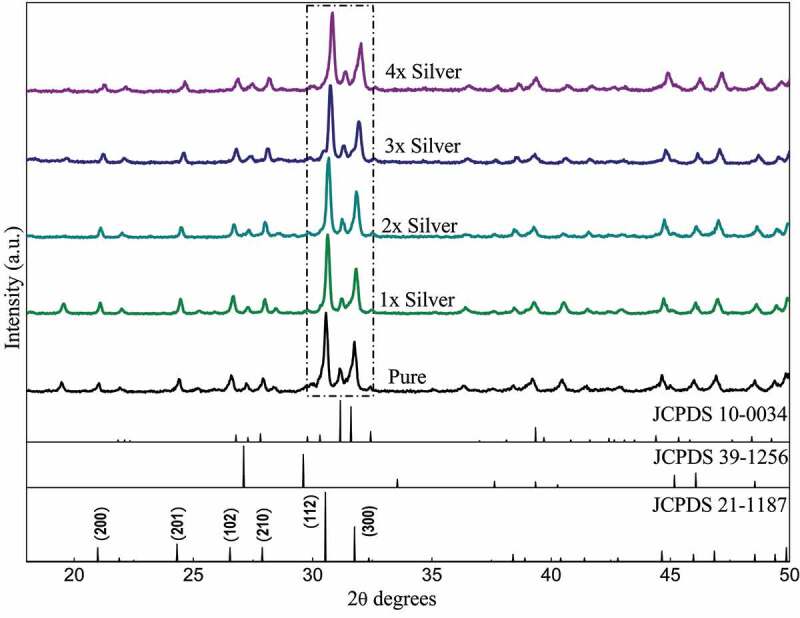
XRD Pattern of pure phase and silver substituted strontium phosphate silicate samples sintered at 800°C

**Figure 1a:**
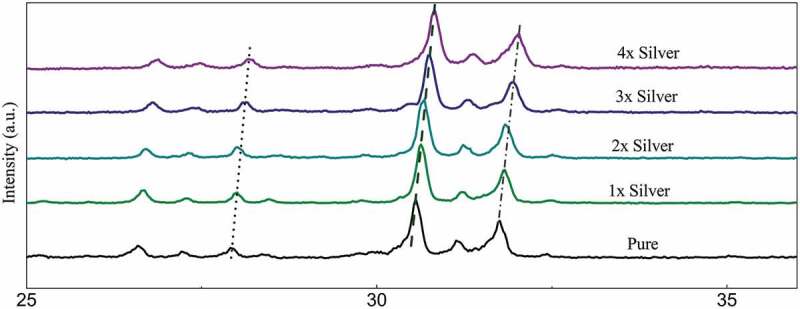
Relative peak shift in diffraction data recorded with substitution of silver in samples sintered at 800°C

**Figure 2:**
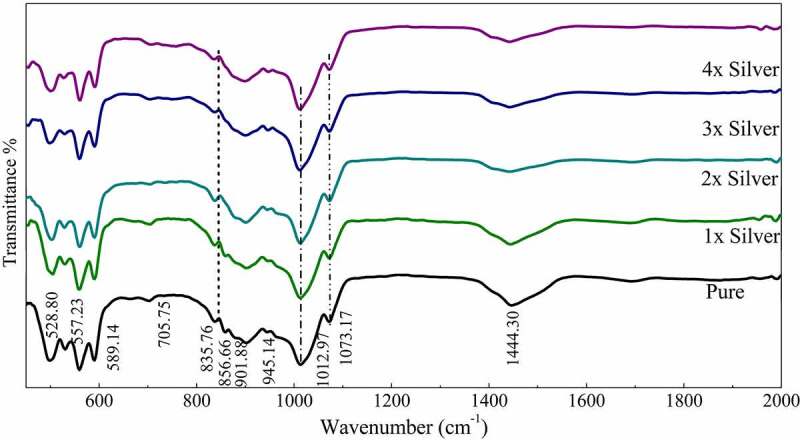
FTIR spectra of pure phase and silver substituted samples sintered at 800°C showing characteristic peaks and bands

FTIR spectroscopy confirmed the formation of pure strontium phosphate silicate with the presence of characteristic phosphate and silicate peaks. The similar position of peaks even with the dopants confirms the structural features even with silver doping and its substitution in the crystal structure [33]. The absorption bands at 1073 (v3), 945 (v1), 589 (v4) and 497 (v2) were detected in the spectrum, were attributed to the phosphate’s (PO_4_^3–^) characteristic absorption and were present throughout the spectra. Other additional absorption bands at 858 cm^−1^ were assigned to the Si-O-Si, detected throughout the pure as well as doped samples [34]. Characteristic vibrations of SiO_2_ (901 and 835 cm^–1^) and CO_3_^2–^ group (1444 cm^−1^) were also recorded. The subsequent increase in silver concentration showed negligible changes in the FTIR spectrum as displayed in the curve (Figure 6). It means an increase in the intensity of the vibration bands of CO_3_^2–^ in the sample as compared to the parent compound. Meanwhile, it is noticed that the stretching vibrations of the phosphorus moiety cover a broader range (1060–1200 cm^–1^), and the region related to the Si-OH band was found to be enhanced with the addition of various concentration of silver [35].

### 3.2. Morphological

**Figure 3.**
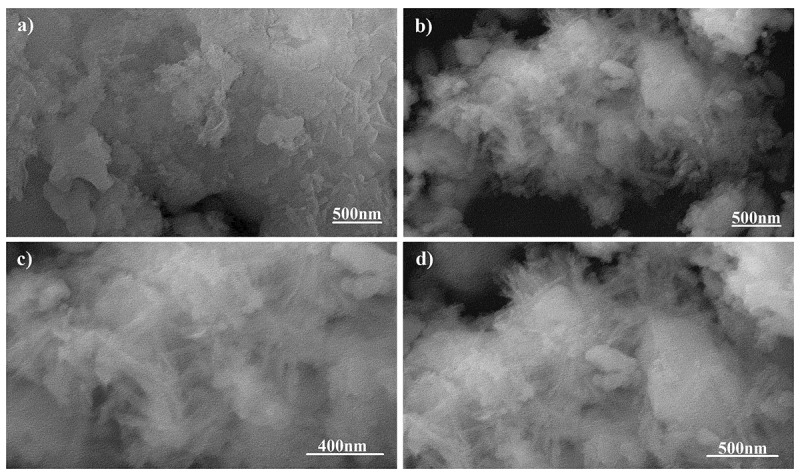
SEM Micrographs of (a) pure Phase and (b) silver substituted samples

The SEM micrographs of pure phase strontium phosphate silicate, as well as silver, substituted samples are almost identical as visible in Figure 3a and 3b respectively. Both the images show a geometric crystalline morphology at a lower magnification that can be attributed to the secondary phases reinforcing the parent material in bulk form. However, an increase in magnification of the doped sample eventually shows the proper needle-shaped morphology of the parent material and confirms the above justification (Figure 3c and 3d).

### 3.3. In-vitro biological evaluations

**Figure 4.**
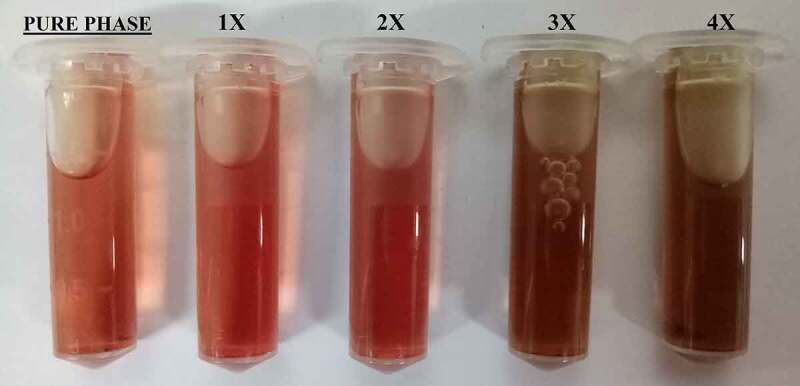
Optical photographs of the pure phase samples by hemolytic assay

**Figure 5a:**
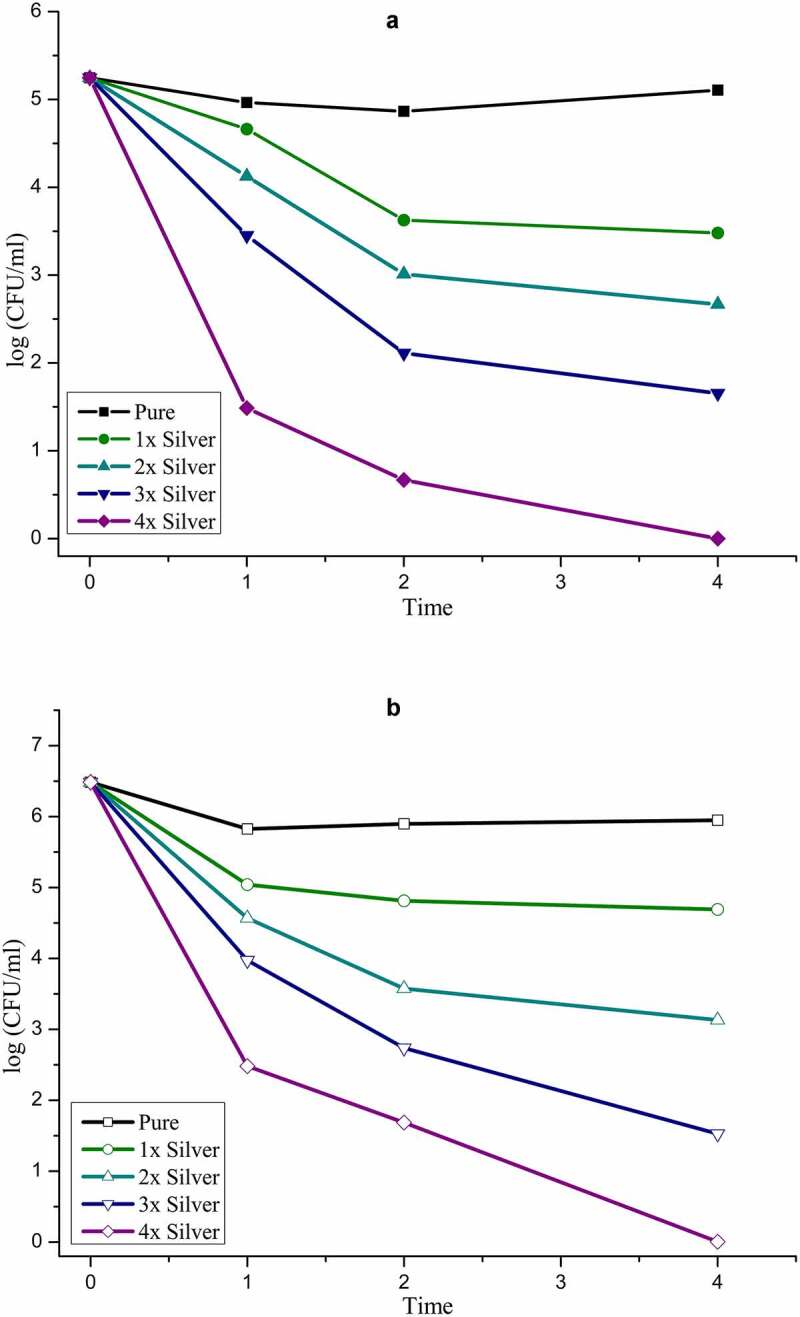
a) Antibacterial effect generated by silver substituted samples over *S. aureus* calculated via CFU. b) Antibacterial effect generated by silver substituted samples over *E. Ccoli* calculated via CFU

Statistical analysis of data obtained from the biological evaluation was performed and studied to evaluate the effects generated by silver substitution. Hemolytic assay of the samples shows proper haemocompatibility at 2% molar substitution as evident from Table 2. An increase in the dopant concentration shows hemolytic effects characterized by the development of dark color after an hour of incubation at body temperature (Figure 4). Studying the data obtained by measuring OD values, samples start showing hemolysis over 2x substitution based on the criterion specified for haemocompatibility [36]. CFU-based quantitative assay of antibacterial effects are plotted in Figure 5 (*S. aureus*: Figure 5a, *E. coli*: Figure 5b), which show an increase in the antibacterial efficacy of doped samples with an increase in dopant concentration. There is a clear difference in the effects generated between the gram-positive and gram-negative species which can be attributed to an additional layer of the cell wall in gram-negative species.

**Table 2.** Absorbance and hemolytic ratio of pure phase and silver substituted samples calculated with respect to OD values

Sample	Absorbance (545 nm)			Hemolysis Ratio			Mean	Std. Dev.
DD Water	1.056	1.056	1.058	Positive Control			_	_
Saline	0.053	0.053	0.053	Negative Control				
Pure	0.067	0.068	0.0067	1.3958	1.4955	1.3958	1.42	0.057
1x	0.07	0.069	0.069	1.6949	1.5952	1.5952	1.63	0.057
2x	0.073	0.072	0.071	1.994	1.8943	1.7946	1.89	0.1
3x	0.077	0.079	0.076	2.3928	2.5922	2.2931	2.42	0.152
4x	0.084	0.087	0.087	3.0907	3.3898	3.3898	3.29	0.172

**Figure 6.**
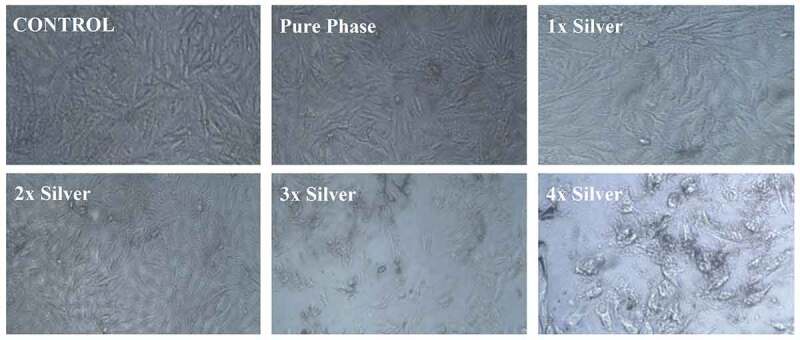
Relative cellular density as observed for different release concentrations of pure phase and silver substituted samples with respect to control

**Figure 7.**
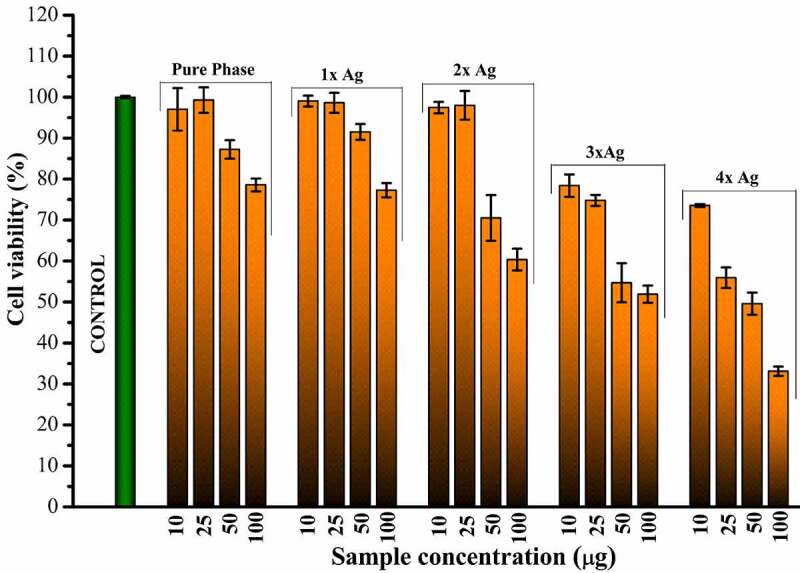
Calculated cell viability as observed for different release concentrations of pure phase and silver substituted samples with respect to control

Cytotoxicity of silver substitution was evaluated using MTT assay of the samples against MG63 cell lines. Optical micrographs of the cellular density show good compatibility of pure phase samples at 25 μg/ml beyond which cell density is found to decrease eventually (Figure 6). This occurs even though there is no cytotoxic component present in the pure phase sample likes of silver as shown in Figure 7. This might be attributed to earlier reports of inhibitory effect toward healthy cells by excess strontium [37]. Considering 25 μg/ml as favorable release concentration, the cytotoxicity data shows 2% molar concentration of silver as the optimum doping to exhibit favorable cell proliferation activity coupled with haemocompatibility and antibacterial effect.

**Figure 8.**
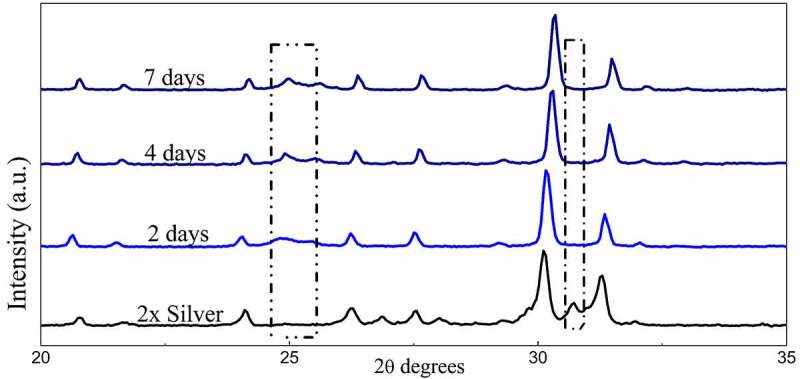
X-ray diffraction pattern of 2x silver substituted samples after simulated body fluid immersion

**Figure 9.**
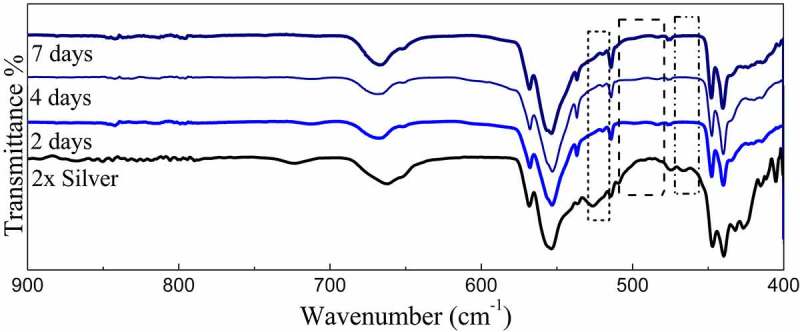
FT-IR Spectra of 2x silver substituted samples after simulated body fluid immersion

**Figure 10.**
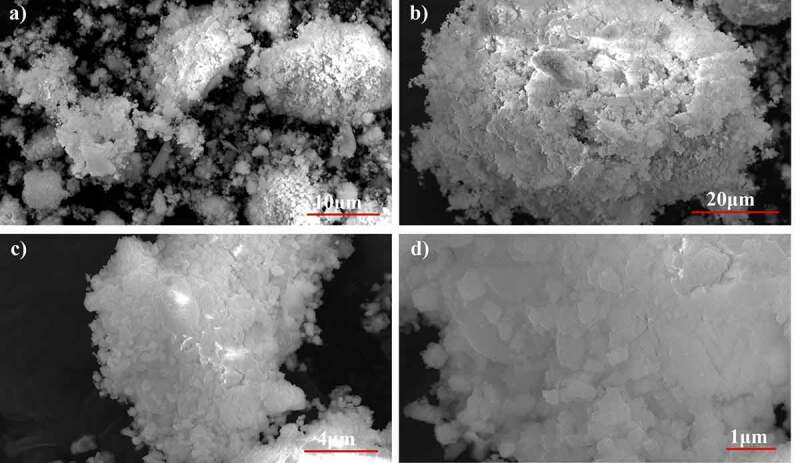
SEM Micrographs of 2x silver substituted samples after simulated body fluid immersion. after 7 days of immersion

Powder X-ray diffraction data of the SBF immersed samples for 4 days and 7 days shows clear loss of secondary phases with an increase in the time frame. Moreover, minor peaks of HAP were discovered which become comparatively prominent with an increase in the treatment period. These minor peaks are marked by a ‘#’ and the peaks of lost secondary phases are indicated by a ‘*’ in Figure 8. IR spectra further confirm the loss of secondary phases of synthesized powders with loss of characteristic phosphate and silicate peaks (Figure 9). However, most of the structural integrity of the parent material seems to be intact and peaks tend to shrink with an increase in the treatment period. The SEM micrographs of samples treated for 7 days show clear differences on the surface morphology of the bulk particles. The bulk particles show dense flake-like outgrowth over the particles otherwise seen with geometric crystalline morphology when untreated (Figure 10a and 10b). The loss of secondary phases and consistent structural integrity of parent material have led to the visualization of distinct needle-shaped apatite more prominently which were otherwise overshadowed by secondary phases and hardly visible as evident from Figure 10c and 10d clearly.

## 4. Discussion

### 4.1 Prologue

The inability to deliver faster bone regeneration rates by calcium phosphate ceramics has led researchers to shift their focus on the discovery of better substitutes. Although these shortcomings have been met in the past with doping in the crystal structure of the ceramics but such doping is often limited in choice due to restrictions in the doping process and the ability of the ceramic to uptake the dopants. The present proposed material with native components that are bioactive as well as stimulate bone regeneration addresses this issue and provides more room for doping apart from the inbuilt feature of stimulated bone growth on account of strontium and silica groups. It is remarkable that the material possesses the same structural features, such as tunnel-shaped structure but has not one but two surfaces with components ready to interact with body fluid that will increase bone formation rates. Literature survey states that several secondary phases, such as strontium phosphate and strontium silicate are present during phase formation of parent material and have a proven track record of aiding osteogenic activity [38,39]. The phase formation studies carried out over strontium phosphate silicate shows 800°C as optimum sintering temperature as major phase formation occurs at 600–650°C retaining these biologically important secondary phases that were confirmed to be useful in SBF treatment studies. Moreover, most of the primary phases are present at this temperature and sharp peaks of the diffraction data also confirm the proper crystallinity of the synthesized powders. [40]

### 4.2 Structural and morphological features

A study of the diffraction pattern shows that all the peaks can be easily assigned to the pure hexagonal bipyramidal phase of Sr_5_(PO_4_)_2_SiO_4_ (ICDD 21–1187) as anticipated [41]. Apart from the designated secondary phases, no other considerable peaks have been observed throughout which establishes the fact that silver has been successfully incorporated in the crystal structure of parent material without any considerable changes [42]. The similarities in ionic radius of strontium and silver facilitates this substitution at strontium sites and the slightly larger ionic radii lead to a shift of major peaks toward greater 2 theta values. A closer look at the shift falling in a straight line shows the agreement between planned and actual product formed in terms of doping concentration.

As of now it is well known that in apatites, silica is substituted at X site which is home of the phosphate group. In this context, an increment in the PO_4_^3 –^ tetrahedral distortion over-stretching vibrations attributed to Si–O–Si bonds appearing in the range of 950–1200 cm^−1^ occurs that is overshadowed by the presence of phosphate groups. However, distinct absorption bands were observed at 856 cm^−1^ assigned to stretching of Si-O-Si [43,44]. Moreover, characteristic vibrations of SiO_2_ were observed at 945 cm^–1^ throughout the spectra. These peaks may or may not be residual precursor, however, it is highly unlikely [45].

Absence of surfactants generally used to separate nanoparticles from each other has led to the agglomeration of 45 nm sized parent material and doped strontium phosphate silicate as observed in high magnification SEM images. Secondary phases act as the reinforcing agents and hide these needle-shaped particles when observed superficially. It is these secondary phases that form the geometrically crystalline shape of bulk particles visible in Figure 3b.

### 4.3 Biocompatibility studies

The in-vitro biological evaluation of the samples was performed dividing the same into three categories that included Biocompatibility evaluation, testing of antibacterial efficacy and observations recorded by in-vitro bone regeneration by treatment of samples to Simulated body fluid.

The biocompatibility studies covered studies on interaction with target cells as well as those analogous to target cells such as MG-63 cell lines. Hemolytic biocompatibility is one of the most important evaluation as blood is the most important component of extracellular matrix and connective tissue at its best often acting as transporter channel. For a material to be viable as an implant, it must be haemocompatible at acceptable component concentrations failing which it might be toxic to our body and healthy cells. This toxicity in our case has been visualized by the darkening of the test samples which are brought about not only by silver as well as parent material due to an unfavorable increase in the pH levels that degenerated the blood cells [46]. This pH upregulation can be attributed to silica phase present as secondary phase as well as a structural component of parent material as silica is known to release alkali ions in the aqueous medium [47]. Since this phenomenon is related to the concentration of material, similar effects were observed in Cytotoxicity studies where a higher release concentration of parent material without any dopant was found to inhibit osteosarcoma cell lines. Moreover, as discussed earlier, a higher concentration of strontium is also to blame for this [48]. However, with cytotoxicity analysis, we can state the favorable release concentration and carry out further studies based on the details. As anticipated, an increase in silver concentration eventually turned toxic toward MG-63 cell lines but at favorable release concentration 2% molar doped samples were found to be optimized and showed better cellular concentration establishing its biocompatibility.

### 4.4 Antibacterial efficacy

Quantitative analysis of antibacterial studies clearly details the antibacterial efficacy of silver substituted samples increasing with an increment of the dopant. The effects were radical with an increase in silver concentration and it is hard to evaluate any other players in the process as silver overshadows them all. However, the concept of pH upregulation brought about by silica components as discussed above must be pointed out as a minor player which may have an effect over Gram-positive bacteria due to the absence of the lipid layer. It was also seen that the effects of silver were comparatively less effective over Gram-negative bacteria which is also a result of the structural features of the cell wall of the bacteria.

### 4.5 In-vitro bone regeneration studies

The available surface area of the material is an important factor in its interaction with body fluids. The native tunnel-shaped structure of P6_3_/m apatites thereby offers the great advantage of exposure to the components accelerating bone regeneration. Diffraction data recorded in Figure 8 shows how secondary phases are lost in the due course of treatment of the powders in SBF. A similar trend is observed in FTIR spectra where the visible loss of phosphate and silicate peaks are observed as compared to the untreated sample along with the shrinking of some peaks. However, even after the loss of secondary phases, it must be noted that major peaks that designate parent material are intact without any changes in both IR as well as diffraction data. Hence, the importance of secondary phases in apatite seeding can be established as the important components that initiate bone formation. The seeded apatites and amorphous calcium phosphates could not be recorded due to very less quantity and their intrinsic properties. This process of apatite seeding has been elaborately discussed by several researchers and the silicate groups are the most important component of the same supported by phosphate groups [40]. The formation of silanol groups forms the base of this operation that attracts oppositely charged calcium ions and forms silicate which later forms phosphates of calcium upon interaction with available phosphate groups [16]. This low Ca–P ratio amorphous phosphate eventually turns into crystalline apatite aided by several metabolic pathways helping in the bone regeneration process [49]. These seeded apatites and Calcium phosphate flakes are clearly visible in SEM images and the degradation of the secondary phase has also exposed the parent material which is now clearly visible otherwise overshadowed by secondary phases.

## 5. Applications and scope

The proposed material might as well be used as bone cements, fillers, etc. It can also be used as an additive to fabricate biphasic bone ceramics and add bioactivity along with predominant strength to bioinert ceramics such as zirconia and alumina. The structural features help in increasing the surface area to interact with fluids and hence coupled with components it will be fruitful to stimulate the bone regeneration process. The research further aims to provide and generate details that may prove as building blocks to choose strontium phosphosilicate over HAP.

## 6. Conclusions

Bulk strontium phosphate silicate was successfully synthesized via solid-state reaction in pure phase as well as with stoichiometric substitution of silver as a dopant using appropriate precursors evident from the diffraction patterns. Silver substitutions show evident changes in diffraction and IR spectra when doped. The antibacterial results to demonstrate the efficacy of silver showed increased cellular apoptosis of bacterial species quantified by CFU-based studies. Biocompatibility studies carried out over hemolytic assay and cytotoxicity assay confirmed 4% molar substitution as the optimized concentration of silver substitution beyond which, toxic effects of silver might harm the native healthy human cells. Simulated body fluid studies showed proper apatite seeding and amorphous HAP was visible as flakes over the bulk particles. Hence, we can state and propose silver substitutes strontium phosphate silicate at 4% molar concentration as a possible and viable candidate to substitute calcium phosphate–based HAP for bone regeneration application with a functional antibacterial property effective against gram-positive as well as gram-negative species.

## Research Highlights

- Evaluating the feasibility of solid-state reaction in preparation of biocompatible ceramics.
- Structural information of silver substituted strontium phosphate silicate
- Morphological features of doped compounds
- Biocompatibility evaluation towards blood cells and MG-63 cell lines.
- Antibacterial activity evaluation of parent material upon substitution of silver.
